# Inhibition of CSF1R, a receptor involved in microglia viability, alters behavioral and molecular changes induced by cocaine

**DOI:** 10.1038/s41598-021-95059-7

**Published:** 2021-08-06

**Authors:** Maria Carolina Machado da Silva, Giovanni Freitas Gomes, Heliana de Barros Fernandes, Aristóbolo Mendes da Silva, Antônio Lúcio Teixeira, Fabrício A. Moreira, Aline Silva de Miranda, Antônio Carlos Pinheiro de Oliveira

**Affiliations:** 1 Neuropharmacology Laboratory, Department of Pharmacology, Universidade Federal de Minas Gerais, Av. Antonio Carlos 6627, Belo Horizonte, MG 31270-901 Brazil; 2 Neurobiology Laboratory Conceição Machado, Department of Morphology, Universidade Federal de Minas Gerais, Belo Horizonte, Brazil; 3 Laboratory of Inflammatory Genes, Department of Morphology, Universidade Federal de Minas Gerais, Belo Horizonte, Brazil; 4 Neuropsychopharmacology Laboratory, Department of Pharmacology, Universidade Federal de Minas Gerais, Belo Horizonte, Brazil; 5 Department of Psychiatry and Behavioral Science McGovern School, The University of Texas Health Science Center at Houston, Houston, USA

**Keywords:** Neuroscience, Reward

## Abstract

Different data suggest that microglia may participate in the drug addiction process as these cells respond to neurochemical changes induced by the administration of these substances. In order to study the role of microglia in drug abuse, Swiss mice aged 8–9 weeks were treated with the CSF1R inhibitor PLX3397 (40 mg/kg, p.o.) and submitted to behavioral sensitization or conditioned place preference (CPP) induced by cocaine (15 mg/kg, i.p.). Thereafter, brains were used to evaluate the effects of CSF1R inhibition and cocaine administration on morphological, biochemical and molecular changes. CSF1R inhibition attenuated behavioral sensitization, reduced the number of Iba-1^+^ cells and increased ramification and lengths of the branches in the remaining microglia. Additionally, both cocaine and PLX3397 increased the cell body to total cell size ratio of Iba-1^+^ cells, as well as CD68^+^ and GFAP^+^ stained areas, suggesting an activated pattern of the glial cells. Besides, CSF1R inhibition increased CX3CL1 levels in the striatum, prefrontal cortex and hippocampus, as well as reduced CX3CR1 expression in the hippocampus. In this region, cocaine also reduced BDNF levels, an effect that was enhanced by CSF1R inhibition. In summary, our results suggest that microglia participate in the behavioral and molecular changes induced by cocaine. This study contributes to the understanding of the role of microglia in cocaine addiction.

## Introduction

According to the World Drug Report 2019^1^, the number of people who used drugs reached 271 million in 2017, with 450,000 deaths in 2015. It is estimated that the use/abuse of drugs generates expenditures of 442 billion dollars annually in the world due costs in the health system, criminal justice and loss of labor productivity^1–3^.

Substance use disorder is defined as a chronic condition characterized by changes in the circuits of reward, motivation and memory systems, and adaptive behavior^4^. Drug abuse can reorganize and promote plastic changes in the reward system and adaptive behavior mainly due to an imbalance in the levels of neurotransmitters in mesocorticolimbic dopaminergic and in corticolimbic glutamatergic pathways^4,5^. However, the etiopathogenesis of addiction is a much more complex process in which people have different susceptibilities^6,7^. In addition, treatment is frequently ineffective and there is an urgent need to better understand the neurobiological mechanisms of addiction to develop novel therapeutic strategies^8^.

Cocaine is one of the most used drugs of abuse worldwide. Its mechanism of action consists of blocking the monoamine membrane transporters of serotonin, norepinephrine and, in particular, dopamine, which increases the availability of these neurotransmitters in the synaptic cleft^9,10^. In addition, cocaine is capable of interacting indirectly with other neuromodulatory circuits, such as glutamatergic, endocannabinoid and GABAergic systems^9,10^. Cocaine also regulates synaptic activities inducing adaptations in memory and learning processes related to positive reinforcement in the reward system^11–13^.

Different studies have suggested that cells of the immune system and glia are related to the neurobiology of addiction^14^. Microglia is the brain resident cell of the immune system and it acts detecting changes in the central nervous system (CNS), releasing inflammatory mediators and phagocytizing cellular debris, eliminating pathogens and tissue debris^15^. In addition, microglia interacts with neurons, astrocytes and oligodendrocytes in a series of processes^16,17^, playing important roles in complex brain functions, such as memory, learning, and adaptive behavior, which are important components in addiction^15,18–22^. These cells act promoting synaptic plasticity^23,24^, at least in part, through the release of neurotrophic factors such as brain-derived neurotrophic factor (BDNF), or cytokines such as tumor necrosis factor (TNF)^21,25^.

Microglia express different receptors and ion channels, commonly found in neurons, which may be the targets of the drugs of abuse. For example, the toll-like receptor 4 (TLR4), which are expressed by immune cells, including microglia, is activated by cocaine^20,26,27^. In addition, the release of dopamine in the nucleus accumbens (NAc), caudate nucleus and putamen^20,28,29^ can activate these cells. In this context, cocaine could induce a chronic and persistent neuroinflammation characterized by changes in the expression of cytokines in the CNS, such as IL-1β, IL-6 and TNF-α^20,26,29,30^, and alterations in the specific CX3CR1-CX3CL1 microglia-neuronal communication pathway^31^.

Pharmacological blocking of microglial activity reverses addiction behaviors induced by different drugs. For instance, treatment with minocycline, a tetracyclic antibiotic able to inhibit microglia, decreased preference induced by cocaine in conditioned place preference (CPP) test^20^, as well as in an alcohol addiction model^32^. Furthermore, the anti-inflammatory and phosphodiesterase inhibitor ibudilast attenuated the behavioral sensitization induced by cocaine^33^. Although these drugs are commonly used to target glia, they have poor selectivity and multiple mechanisms of action. For example, minocycline potentiates the phosphorylation of GluA1 subunit of the AMPA receptor, as well as decreases glutamatergic calcium signaling and may change key processes of memory and learning^34,35^. Furthermore, inhibition of phosphodiesterase 4 enzyme, the target of ibudilast, reverses behavioral sensitization, in addition to restoring the balance between inhibitory and excitatory pathways after cocaine administration^36^.

PLX3397 is an inhibitor of the colony stimulating factor 1 receptor (CSF1R), which is essential for microglial viability. The depletion of these cells by PLX3397 is an efficient method for studying the microglial functions, without promoting cognitive and behavioral changes^31,37^. Considering previous evidence suggesting a potential role of microglia in the neurobiology of addiction^20,29^, we tested the hypothesis that CSF1R inhibition attenuates cocaine-induced behavioral changes by regulating brain immune responses.

## Results

### CSF1R inhibition decreased behavioral sensitization, but not CPP, induced by cocaine

Behavioral sensitization and CPP models are capable of mimicking the plastic changes that occur in the transition from a pattern of recreational use to a pattern of abusive use that characterizes addiction^38^. To verify whether microglia participate in neurobiological changes induced by cocaine, animals were treated with the CSF1R inhibitor drug PLX3397.

**Figure 1:**
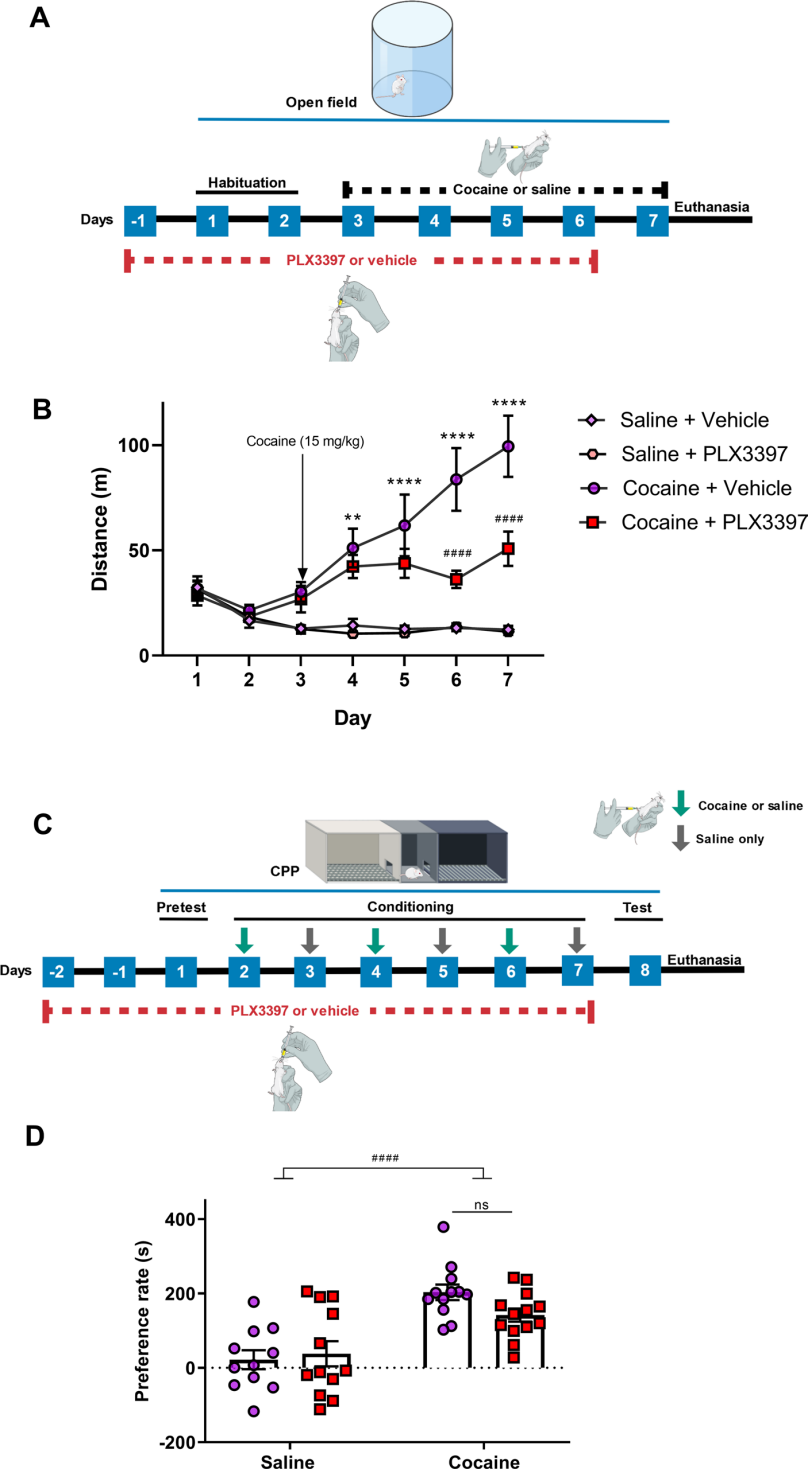
Effect of partial microglia depletion on the behavioral sensitization and conditioned place preference induced by cocaine. Experimental design of behavioral sensitization (**A**). Daily distance travelled by animals injected with cocaine or saline in the behavioral sensitization test [treatment factor (F_(3,24)_ = 18.85; p < 0.0001), time factor (F_(4,96)_ = 15.59; p < 0.001), interaction (F_(12,96)_ = 9.72; p < 0.0001)] (n = 6–8 in each group) (**B**). Experimental design of conditioned place preference (**C**). Rate of preference in the conditioned place preference test [cocaine factor (F_(1,45)_ = 34.32; p < 0.0001), CSF1R inhibition factor (F_(1,45)_ = 0.29; p = 0.5916) and cocaine vs CSF1R inhibition factor (F_(1,45)_ = 3.36; p = 0.0734)] (n = 11–13 in each group) (**D**). Results are expressed as mean ± SEM. ^#^p < 0.05 difference between the saline and cocaine groups; ***p < 0.001 and ****p < 0.0001 compared with the PLX3397 treated group.

Cocaine increased the locomotor activity of the animals over the days (behavioral sensitization) (p = 0.0029 among cocaine + vehicle vs saline + vehicle). CSF1R inhibition reduced behavioral sensitization induced by cocaine, starting on the fourth day (p < 0.0001 among cocaine + vehicle and cocaine + PLX3397) (Fig. 1B). There was no statistical difference between control group and group treated only with PLX3397, which demonstrates that this drug does not influence basal locomotor activity of the animals.

In the CPP test, cocaine increased the rate of preference for the compartment where the animals received cocaine (i.e. drug-paired side) (p < 0.0001). CSF1R inhibition was not able to reverse this preference (Fig. 1D). All animals were weighted every 2 days during behavioral assessment. Neither cocaine nor PLX3397 changed the body mass of the animals over the days of treatment (data not shown).

### CSF1R inhibition and cocaine induced microglial alterations in NAc

**Figure 2:**
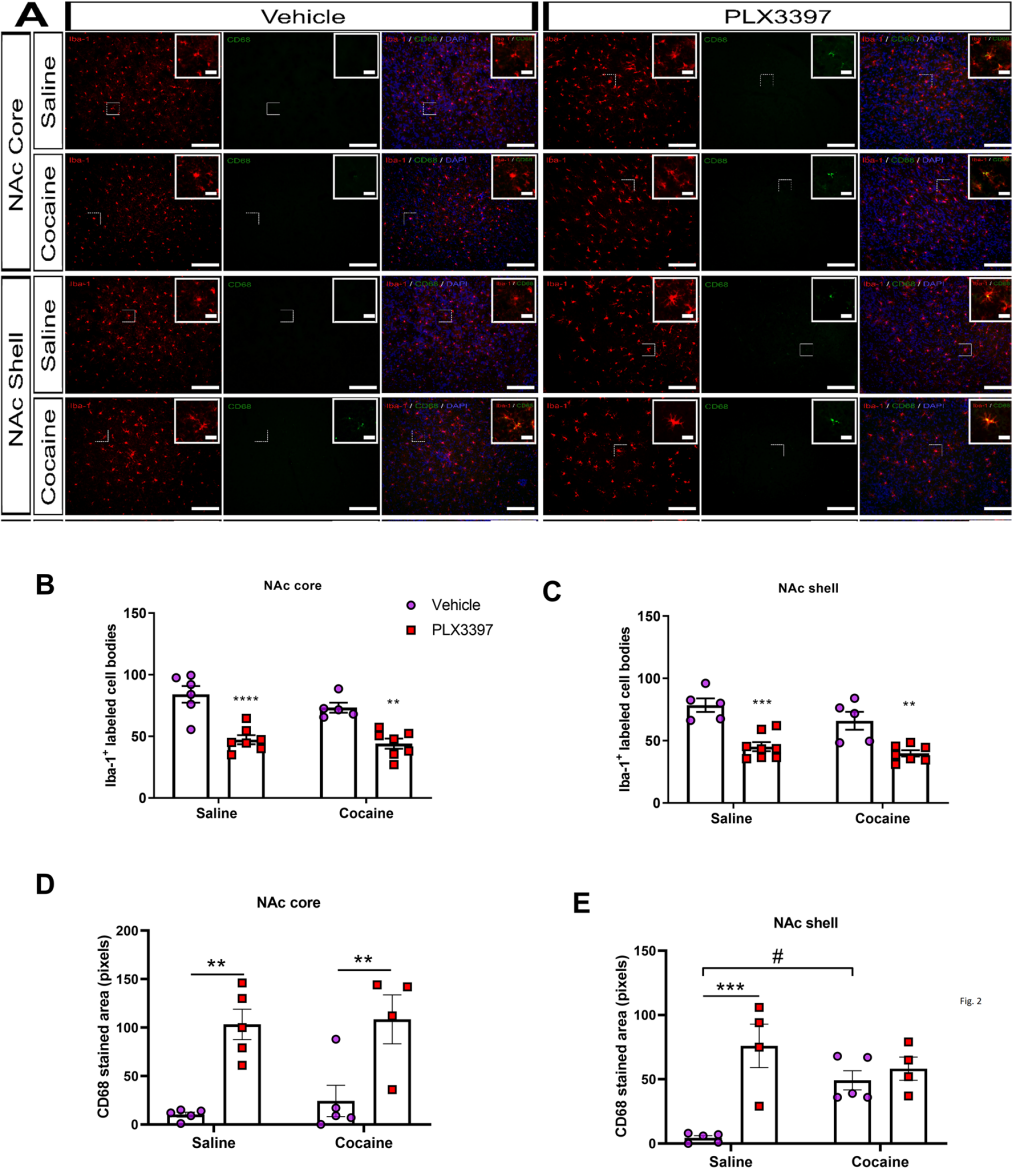
Effects of CSF1R inhibition and cocaine administration on the number of Iba-1^+^ cells and CD68^+^ stained area. Representative images of the number and activation of microglia in the NAc core and NAc shell (**A**). Number and activation of microglial cells in the NAc core [cocaine factor (F_(1,21)_ = 2.15; p = 0.1566), CSF1R inhibition factor (F_(1,21)_ = 47.37; p < 0.0001) and cocaine vs CSF1R inhibition factor (F_(1,21)_ = 0.63; p = 0.4358)] and [cocaine factor (F_(1,15)_ = 0.36; p = 0.5540), CSF1R inhibition factor (F_(1,15)_ = 30.92; p < 0.0001) and cocaine vs CSF1R inhibition factor (F_(1,15)_ = 0.07; p < 0.07887)] for Iba-1^+^ cells and CD68^+^ stained area, respectively (**B**, **D**); NAc shell [cocaine factor (F_(1,21)_ = 3.84; p = 0.0633), CSF1R inhibition factor (F_(1,21)_ = 42.44; p < 0.0001)], and cocaine vs CSF1R inhibition factor (F_(1,21)_ = 0.59; p = 0.4480)] and [cocaine factor (F_(1,14)_ = 2.04; p = 0.1750), CSF1R inhibition factor (F_(1,14)_ = 18.14; p = 0.0008) and cocaine vs CSF1R inhibition factor (F_(1,14)_ = 10.91; p = 0.0052)] for Iba-1^+^ cells and CD68^+^ stained area, respectively (**C**, **E**); of animals submitted to the behavioral sensitization. Microscope lens 20 × and 125 µm scale bar for the images, and microscope lens 40 × and 25 µm scale bar for the inserts. Results are expressed as mean ± SEM. #p < 0.05 difference between saline and cocaine groups. **p < 0.01 and ***p < 0.001 compared with the PLX3397 treated group (n = 5–7 in each group).

To verify whether there was a decrease of microglia number, Iba-1^+^ cells were evaluated in the NAc core and shell. CSF1R inhibition decreased the number of Iba-1^+^ cells in the Nac core in both saline and cocaine treated groups (p < 0.0001). Cocaine per se did not change the number of these cells (p = 0.1566) (Fig. 2A,B). Similarly, animals treated with PLX3397 revealed a reduced number of Iba-1^+^ cells in the NAc shell (p < 0.0001) (Fig. 2A,C).

In order to evaluate whether the treatment with PLX3397 and cocaine would induce microglia activation, we performed immunostaining for CD68. This transmembrane glycoprotein is expressed by the microglia and suggests a phagocytic activity^39^. In the NA core, only the CSF1R inhibition induced an increase in CD68^+^ (p < 0.0001) (Fig. 2A,D). On the other hand, both cocaine and CSF1R inhibition increased CD68 stained area in the NAc shell (p = 0.0008 and p = 0.0052) (Fig. 2A,E).

**Figure 3:**
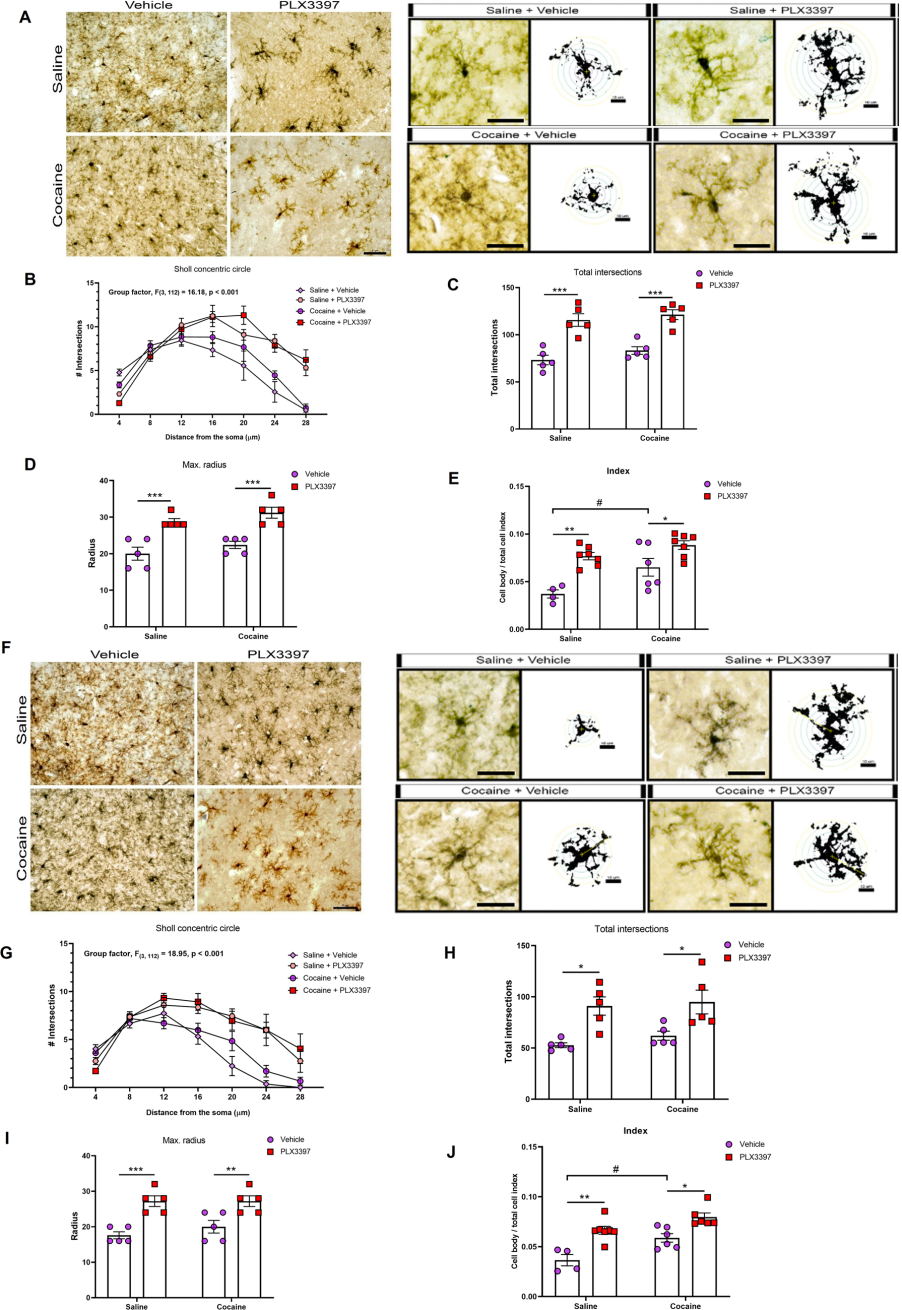
Effects of CSF1R inhibition and cocaine on microglia morphology. Representative images of the microglia morphology in the NAc core (**A**) and NAc shell (**F**). Quantification of microglia branches ramification [NAc core—radius factor (F_(6,112)_ = 45.30; p < 0.0001), treatment factor (F_(3,112)_ = 16.18; p < 0.0001) and interaction factor (F_(18,112)_ = 4.56; p < 0.0001); NAc shell—radius factor (F_(6,112)_ = 6.11; p < 0.0001), treatment factor (F_(3,112)_ = 18.95; p < 0.0001) and interaction factor (F_(18,112)_ = 3.16; p < 0.0001)] (**B**, **G**), total number of intersections [NAc core—cocaine factor (F_(1,16)_ = 2.203; p = 0.1571), CSF1R inhibition factor (F_(1,16)_ = 56.83; p < 0.0001) and cocaine vs CSF1R inhibition factor (F_(1,16)_ = 0.16, p = 0.6926); NAc shell—cocaine factor (F_(1,16)_ = 0.7361; p = 0.4036), CSF1R inhibition factor (F_(1,16)_ = 21.20; p < 0.0003) and cocaine vs CSF1R inhibition factor (F_(1,16)_ = 0.11, p = 0.7365)] (**C**, **H**), total branch length (max. radius) [NAc core—cocaine factor (F_(1,16)_ = 3.273; p = 0.0893), CSF1R inhibition factor (F_(1,16)_ = 44.00; p < 0.0001) and cocaine vs CSF1R inhibition factor (F_(1,16)_ = 0.00, p = 0.999); NAc shell—cocaine factor (F_(1,16)_ = 0.6667; p = 0.4262), CSF1R inhibition factor (F_(1,16)_ = 32.67; p < 0.0001) and cocaine vs CSF1R inhibition factor (F_(1,16)_ = 0.66, p = 0.4262)] (**D**, **I**) and cell body to total cell size ratio [NAc core—cocaine factor (F_(1,20)_ = 10.48; p = 0.0041), CSF1R inhibition factor (F_(1,20)_ = 26.42; p < 0.0001), and cocaine vs CSF1R inhibition factor (F_(1,20)_ = 1.76; p = 0.1985)] [NAc shell—cocaine factor (F_(1,19)_ = 15.76; p < 0.001), CSF1R inhibition factor (F_(1,19)_ = 32.27; p < 0.0001) and cocaine vs CSF1R inhibition factor (F_(1,19)_ = 1.03, p = 0.3225)] (**E**, **J**) in the NAc core and NAc shell, respectively. Microscope lens 40 × and 25 µm scale bar for the images, and 25 µm scale bar for the two-dimensional representation. Results are expressed as mean ± SEM. ^#^p < 0.05 difference between saline and cocaine groups. *p < 0.05, **p < 0.01, ***p < 0.001 and ****p < 0.0001 compared with the PLX3397 treated group (n = 4–5 in each group).

As alterations in microglial morphology may suggest changes in microglial function^15,40,41^, we applied Sholl analysis and cell body to total cell size ratio^42–44^ in order to evaluate the effects of CSF1R inhibition or cocaine under microglial morphology. Both cocaine and PLX3397 increased microglial activation index in the NAc core (p = 0.0041 and p < 0.0001) (Fig. 3A,E) and in the NAc shell (p < 0.001 and p < 0.0001) (Fig. 3F,J). Sholl analysis revealed a significant effect of CSF1R inhibition on the morphology of microglia. In general, microglia from PLX3397-treated mice presented more ramified branches (p < 0.0001 and p < 0.0001) (Fig. 3A,B,F,G). The CSF1R inhibitor, but not cocaine, increased the total number of intersections (p < 0.0001 and p < 0.0003) and the total branch length (max. radius) (p < 0.0001 and p < 0.0001) of remaining microglia from NA core (Fig. 3A,C,D) and NAc shell (Fig. 3F,I,H).

### Association of cocaine and CSF1R inhibition altered CX3CR1 expression and CX3CL1 levels

**Figure 4:**
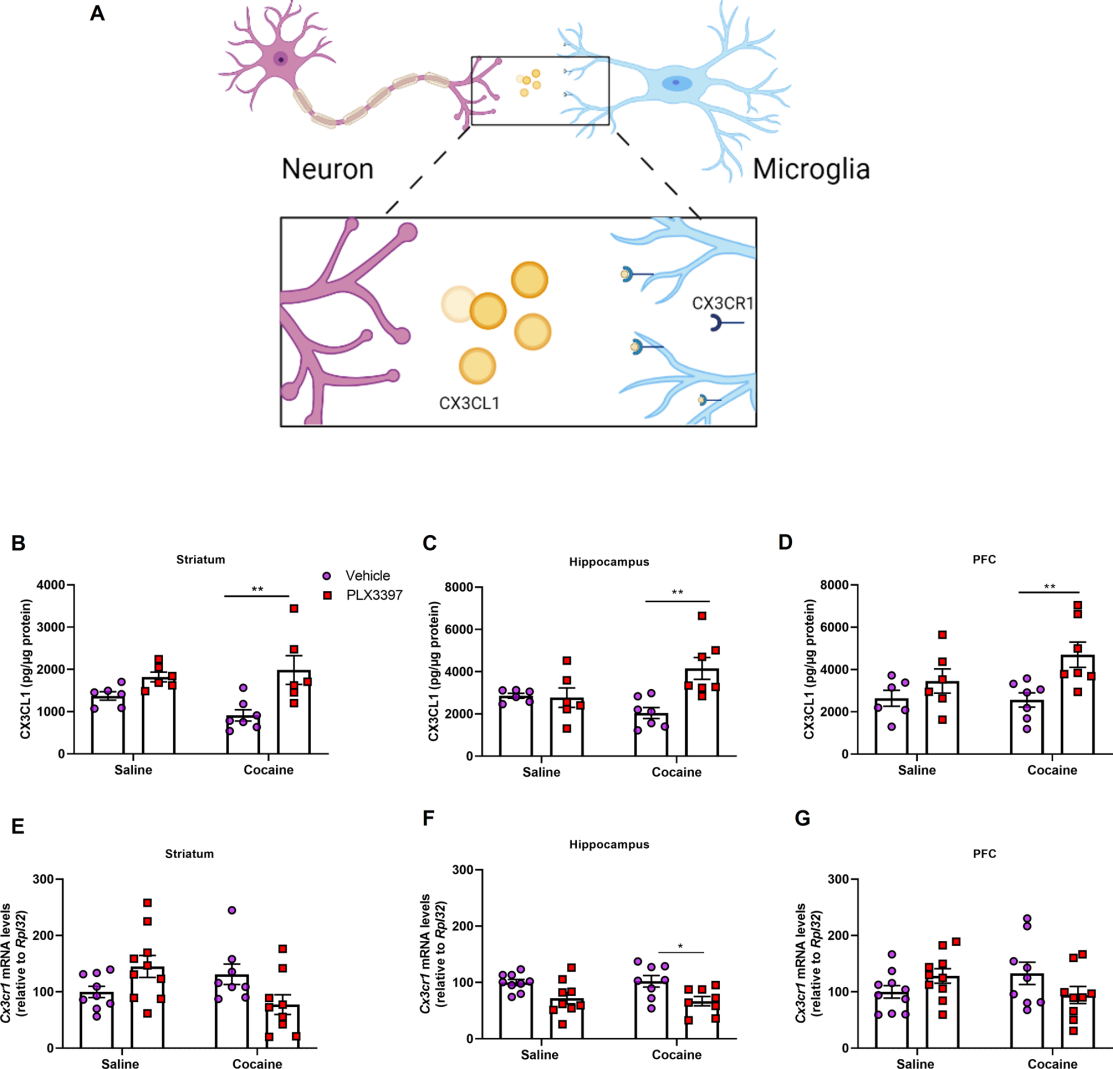
Effect of CSF1R inhibition and cocaine administration on CX3CL1 levels and CX3CR1 expression in the brain. Representative image of the CX3CL1-CX3CR1 pathway (**A**). Quantification of CX3CL1 in the striatum [cocaine factor (F_(1,21)_ = 0.58; p = 0.4526), CSF1R inhibition factor (F_(1,21)_ = 15.35; p = 0.0008) and cocaine vs CSF1R inhibition factor (F_(1,21)_ = 2.58; p = 0.1230)] (**B**), hippocampus [cocaine factor (F_(1,22)_ = 0.55; p = 0.4641), CSF1R inhibition factor (F_(1,22)_ = 6.93; p = 0.0152) and cocaine vs CSF1R inhibition factor (F_(1,22)_ = 8,23; p = 0.0089)] (**C**) and PFC [cocaine factor (F_(1,22)_ = 1.42; p = 0.2456), CSF1R inhibition factor (F_(1,22)_ = 9.17; p = 0.0062) and cocaine vs CSF1R inhibition factor (F_(1,22)_ = 1.82; p = 0.1905)] (**D**) (n = 5–7 in each group). Quantification of CX3CR1 in the striatum [cocaine factor (F_(1,32)_ = 1.16; p = 0.2896), CSF1R inhibition factor (F_(1,32)_ = 0.06; p = 0.8031) and cocaine vs CSF1R inhibition factor (F_(1,34)_ = 5.08; p = 0.0307) (**E**), hippocampus [cocaine factor (F_(1,30)_ = 0.03; p = 0.8522), CSF1R inhibition factor (F_(1,30)_ = 13.03; p = 0.0011) and cocaine vs CSF1R inhibition factor (F_(1,30)_ = 0.18; p = 0.6716)] (**F**) and PFC [cocaine factor (F_(1,34)_ = 0.00; p = 0.97), CSF1R inhibition factor (F_(1,34)_ = 0.11; p = 0.7390) and cocaine vs CSF1R inhibition factor (F_(1,32)_ = 8.57; p = 0.0062) (**G**) (n = 8–10 in each group). Results are expressed as mean ± SEM. *p < 0.05 and **p < 0.01 compared with the PLX3397 treated group.

The chemokine CX3CL1, also known as fractalkine, together with its receptor CX3CR1, is important for microglia-neuron signaling (Fig. 4A), regulating, among others, synaptic plasticity and transmission, neuronal development and maturation and neurogenesis^21,45,46^. Thus, considering that some of these phenomena are important for addiction, we evaluated whether this pathway would be altered by cocaine and inhibition of CSF1R.

CSF1R inhibition increased CX3CL1 levels in the striatum (p = 0.0008) and in the PFC (p = 0.0062) (Fig. 4B,D). In the hippocampus, PLX3397 increased the CX3CL1 concentration only in the animals treated with cocaine (p = 0.0152 and p = 0.0089 for cocaine factor and interaction factor, respectively) (Fig. 4C).

We further measured the mRNA levels of CX3CR1 (*Cx3cr1*) in the hippocampus, PFC and striatum. In the hippocampus, CX3CR1 mRNA levels were decreased by CSF1R inhibition (p = 0.0011) (Fig. 4F). In the striatum and PFC, an interaction was observed (p = 0.0307 and p = 0.0062 for striatum and PFC, respectively) suggesting that PLX3397 induced opposite effects in animals treated with saline and cocaine (Fig. 4E,G).

### Cocaine and CSF1R inhibition altered BDNF levels and TrkB expression

**Figure 5:**
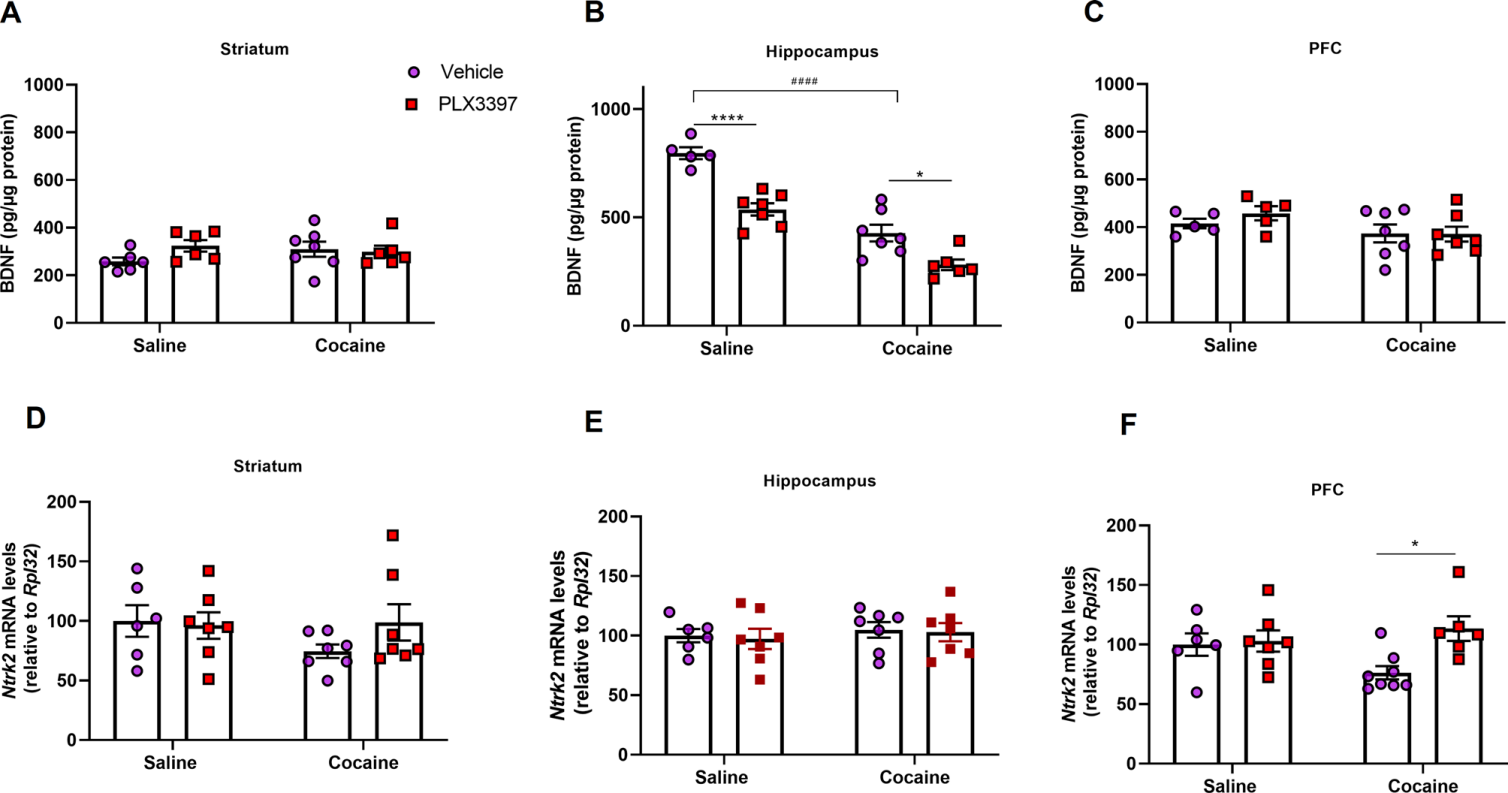
Effect of CSF1R inhibition and cocaine administration on neurotrophic levels and TrkB mRNA expression in the brain. Quantification of BDNF in the striatum [cocaine factor (F_(1,21)_ = 0.27; p = 0.6058), CSF1R inhibition factor (F_(1,21)_ = 1.15; p = 0.2946) and cocaine vs CSF1R inhibition factor (F_(1,21)_ = 2.17; p = 0.1555) (**A**), in the hippocampus [cocaine factor (F_(1,21)_ = 97.38; p < 0.0001), CSF1R inhibition factor (F_(1,21)_ = 41.00; p < 0,0001) and cocaine vs CSF1R inhibition factor (F_(1,21)_ = 3.22; p = 0.0868)] (**B**) and in the PFC [cocaine factor (F_(1,20)_ = 3.81; p = 0.0650), CSF1R inhibition factor (F_(1,20)_ = 0.37; p = 0.5455) and cocaine vs CSF1R inhibition factor (F_(1,20)_ = 0.48; p = 0.4937)] (**C**). Quantification of TrkB mRNA expression in the striatum [cocaine factor (F_(1,23)_ = 0.93; p = 0.34), CSF1R inhibition factor (F_(1,23)_ = 0.75; p = 0.3940) and cocaine vs CSF1R inhibition factor (F_(1,23)_ = 1.41; p = 0.2458)] (**D**), hippocampus [cocaine factor (F_(1,23)_ = 0.51; p = 0.4797), CSF1R inhibition factor (F_(1,23)_ = 0.10; p = 0.7518) and cocaine vs CSF1R inhibition factor (F_(1,23)_ = 0.003; p = 0.9541)]. (**E**) and PFC [cocaine factor (F_(1,23)_ = 0.62; p = 0.4371), CSF1R inhibition factor (F_(1,23)_ = 5.52; p = 0.0265) and cocaine vs CSF1R inhibition factor (F_(1,23)_ = 4.04; p = 0.0563)] (**F**). Results are expressed as mean ± SEM. ^#^p < 0.05, ^##^p < 0.01 and ^####^p < 0.0001 difference between the saline and cocaine groups. *p < 0.05 and ****p < 0.0001 compared with the PLX3397 treated group (n = 4–7 in each group).

To better understand the role of microglia in neuroplastic changes induced by cocaine, we evaluated the BDNF levels, and TrkB (*Ntrk2*) expression in the striatum, hippocampus and PFC. Cocaine reduced the BDNF levels in the hippocampus. Besides, CSF1R inhibition decreased BDNF levels both in saline or cocaine groups (p < 0.0001, p < 0.0001 and p = 0.0868 for cocaine factor, CSF1R inhibition factor and interaction, respectively) (Fig. 5B). In the PFC, there was a trend towards a decrease in the BDNF levels of the groups treated with cocaine in comparison with the saline groups (p = 0.0650). There was no difference in the BDNF levels in the striatum (Fig. 5A,C).

CSF1R inhibition prevented the decrease in TrkB mRNA levels in the PFC induced by cocaine (p = 0.0265 and p = 0.0563) (Fig. 5F). However, no difference in TrkB mRNA levels was observed between the groups in the hippocampus and in the striatum (Fig. 5D,E).

In order to further investigate the effect of cocaine and CSF1R inhibition in the production of neurotrophic factors, we evaluated GDNF and NGF. Cocaine decreased GDNF and NGF levels in the PFC. However, neither cocaine nor CSF1R inhibition altered GDNF and NGF in the other regions (Supplementary Table 1).

### Cocaine and CSF1R inhibition increased GFAP stained area in the NA

Once both astrocytes and microglia establish a reciprocal modulation of their functions, and cocaine induces astrogliosis^47,48^, GFAP-immunolabeling was performed to quantify these cells in the NAc core and shell. In both regions, cocaine increased GFAP^+^ (p < 0.001 and p = 0.0010) cells area. PLX3397 also increased GFAP-immunostaining in comparison with the saline group in the NAc core (p < 0.0001 and p = 0.005 for CSF1R inhibition factor and interaction, respectively) and in the NAc shell (p < 0.0001 and p < 0.05 for CSF1R inhibition factor and interaction, respectively) (Supplementary Fig. S1).

### CSF1R inhibition and cocaine also induced microglial alterations in CA1

Since CA1 hippocampal region receives dopaminergic innervation from VTA and projects glutamatergic fibers into the NAc^12^, and we found important alterations in CX3CL1-CX3CR1 and BDNF signaling in the hippocampus, we decide to investigate histological alterations in the microglia from CA1. PLX3397 decreased the number of Iba-1^+^ cells in the CA1 region (p < 0.0001). Furthermore, both cocaine and CSF1R inhibition increased CD68 stained (p = 0.0130 and p = 0.00210). CSF1R inhibition increased microglial activation index (p < 0.01), the total radius (p < 0.0001) and the total branch length (p = 0.0004). Finally, both cocaine and CSF1R inhibition increased the total intersection (p < 0.001 and p < 0.0001) (Supplementary Fig. S2).

## Discussion

There is evidence that immune and glial cells, such as microglia, are implicated in the neurobiology of addiction^29,31^. In this study, by blocking the CSF1R, we induced alterations in microglia morphology and changes in CX3CL1-CX3CR1 and BDNF-TrkB levels in cocaine treated animals, which could be related to the protective effect of PLX3397 in behavioral sensitization induced by cocaine.

PLX3397 is a highly selective CSF1R/c-Kit inhibitor that has been used to evaluate the role of microglia in various conditions^49^. Off-target effects seem unlikely due to the lower affinity of the inhibitors to other kinases^50^. Therefore, the results of this and other studies are strongly associated with CSF1R inhibition and microglia depletion.

We found that CSF1R inhibition reduced behavioral sensitization induced by cocaine. This finding is in line with previous studies that used the drugs ibudilast and minocycline, capable of blocking the activation of Iba-1^+^ cells, to attenuate cocaine-induced behavioral sensitization^33,51^. Similarly, PLX3397 also blocked nicotine induced increase in cocaine reinforcement^31^. These studies suggest that the activation of microglia in the striatum activates dopaminergic neurons that could modulate the rewarding effects of cocaine^20,31^.

On the contrary, treatment with PLX3397 did not alter the CPP induced by cocaine. Similarly, previous studies have demonstrated that a reduction of the number or activity of microglia with minocycline, GW2580 and mac-1-saporin also did not alter the behavior in this model^52,53^. However, Northcutt et al. demonstrated that minocycline inhibits cocaine-induced CPP in rats^20^. These discrepancies are not clear due to the mechanism of action of minocycline, since this drug modulates in different ways the glutamatergic pathway, inhibits metalloproteinases, inhibits apoptosis, among other effects^54^. The behavioral sensitization enables the study of the development of neural changes induced by the repeated treatment with different drugs, while the CPP is used for assessing the learning and memory associated with the context induced by drug rewarding effects. These studies highlight the complexity of the neurobiology of addiction, a complex process that recruits different neural pathways and plastic processes, such as associative and non-associative sensitizations^55,56^.

In a previous study, PLX3397 treatment of C57Bl/6 mice for 21 days was sufficient for a robust microglial depletion^37^. In the present study, we treated the Swiss mice for 7 days and found a depletion of around 50% of the microglia in the NAc core, NAc shell and CA1. In agreement with our study, other studies observed only a partial microglia reduction using the PLX3397 at the same doses for 7 days or more^57–59^. PLX5622, another CSF1R inhibitor, also depleted 30% of microglia after 7^60,61^ and 21^60^ days of treatment. Thus, in studies aiming at the depletion of microglia, different factors, such as administration protocol, animal strain and the type of inhibitor, should be considered in order to completely or partially reduce the cell population.

The reduction in microglia population is an important protocol to understand the role of these cells in different pathophysiological processes. Partial microglia depletion induced by PLX3397 was used to study the role of these cell in animal models of depression^62^, viral-induced neurologic damage^58^, tau pathology^57^, behavioral and neuronal alterations induced by lipopolysaccharide^63^, autoimmune encephalomyelitis^64^ and amyotrophic lateral sclerosis^59^. Besides, partial microglia depletion induced by other drugs such as GW2580, liposome-encapsulated clodronate and BLZ945 was also used to understand the role of these cells in spinal cord injury^65^, remyelination process^66^, neuropathic pain^67^, as well as the stress-induced susceptibility to CPP induced by cocaine^53^. Considering the immune role of microglia, the entire depletion of these cells in a non-sterile condition could induce a transient immunodeficiency and facilitate the appearance of infections^49^.

Similar to our study, previous reports have shown the beneficial effects of CSF1R inhibition in models of brain disorders^59,63,64^. These protective effects induced by CSF1R inhibition could be due to different mechanisms. On one hand, the remaining cells could present a modified activity that would avoid the behavioral impairments induced by cocaine. In fact, it has been shown that the remaining microglia are less inflammatory in protocols of CSF1R inhibition^64,68^, while cocaine induces a more pro-inflammatory microglia profile^29,30^. On the other hand, the beneficial effect observed with the CSF1R inhibition could be due to the reduction of the viable cells that could contribute to behavioral sensitization.

CSF1R inhibition also promoted morphological changes in the remaining microglia, as revealed by the increased ramification and lengths of the branches, and an increase in cell body size. In addition, we noticed an increased CD68-stained area in both PLX3397 and cocaine-treated mice, suggesting an altered microglial state of activation in animals submitted to CSF1R inhibition or cocaine exposition. In agreement with our data, Bennet et al. and Spiller et al. also observed an upregulation of CD68 in animals treated with PLX3397^57,59^. Although cocaine did not alter the estimated number of Iba-1^+^ cells in the NAc, we noticed an increase in the cell body to total cell size ratio in the core and shell in this region of animals treated with cocaine. This suggests that cocaine activates microglia in these brain regions, as demonstrated previously^20,29^. One possible mechanism that could explain the activation of these cells by cocaine is through the activation of TLR4. Cocaine is capable of binding to the MD-2/TLR4 complex in microglia, being recognized as an exogenous substance, which leads to a neuroinflammatory response^20,26,27,29^.

Notwithstanding both cocaine and PLX3397 altered the morphology of microglia that resembles an activated state, it is worth noting that the activity of the cells induced by both stimuli may differ. Indeed, microglia possess a wide spectrum of phenotypes^69^. The microglia from animals treated with PLX3397 plus cocaine presents a different morphology from animals treated with only cocaine, with increased ramification and lengths of the branches, and larger cell body. Importantly, morphological modifications are known to be related to alterations in microglia function^15,40,41^, which suggests that the remaining cells are likely playing a different role in the NAc and in the hippocampus of PLX3397-treated mice.

The CX3CL1-CX3CR1 pathway is important for neuroinflammation, synaptic plasticity and neurogenesis^45,70^. We found that CSF1R inhibition increased CX3CL1 in the group treated with cocaine, but not with saline. In accordance with our study, Zhang et al. also demonstrated that PLX3397 per se did not alter the CX3CL1 levels^71^. It has been shown that, in an environment with microglia depletion, a TLR4 agonist induces a higher expression of CX3CL1 mRNA in comparison with the activation of the receptor in a tissue containing a normal glial cell population^72^. Importantly, cocaine may also bind and activate microglial TLR4^20^. This also could, at least partially, explain the increase in CX3CL1 in the cocaine + PLX3397 group.

Here, CX3CR1 was reduced in the hippocampus of mice treated with CSF1R inhibitor and cocaine in comparison with the animals treated only with cocaine. It has been demonstrated that no reduction in the expression of the receptor is observed with a 30% depletion of microglia cells^57^. On the other hand, the depletion of 90% of the cells with PLX3397 decreased CX3CR1 expression^72,73^. Thus, considering that CX3CR1 is expressed by microglia, a decrease in the amount of the receptor expression in the tissues may be due to the reduction of the proportion of these cells. Besides, the expression of the receptor could be differently regulated depending on the brain region, due to the neurotransmitter and cytokines milieu present in the area, as well as the stimulus used^74,75^.

Although the CX3CR1/CX3CL1 pathway is important for neuron-microglia communication in both physiological and pathological conditions, only few studies have investigated its role in animal models of addiction. For example, morphine treatment did not alter the expression of CX3CR1, but decreased CX3CL1 levels^76^. Linker et al. showed that CX3CL1 is required for the increase in cocaine self-administration and microglial activation induced by nicotine in adolescent mice^31^. Finally, CX3CR1-deficient mice revealed impaired extinction of cocaine induced-CPP, but not acquisition and reinstatement^77^.

In the present study, both cocaine and microglia depletion reduced the levels of BDNF in the hippocampus. In agreement with our results, it has been shown that repeated administration of cocaine leads to a reduction of this neurotrophic factor in the hippocampus^78,79^. Although microglia may not represent the main source of BDNF in the brain, the release of this neurotrophin by these cells seems to be important in physiological and pathological conditions^80^. In addition, it has been shown that BDNF synthesis and release are regulated by a plethora of events and circumstances, such as altered levels of ions, depolarization, neurotransmitters, hormones, inflammatory mediators, among others^81^. In this sense, alteration of microglia population and properties may alter the hippocampal environment, leading to varied stimuli that could influence the production of this neurotrophic factor, such as neurons and oligodendrocytes. This could partially explain the results obtained in the present study. However, the reduction of BDNF induced by cocaine and/or microglia depletion, as well as its role in sensitization induced by cocaine, should be investigated in future studies.

As previously mentioned, the role of TrkB-BDNF signaling may vary depending on the region in the context of drug addiction^82–85^, although this is not fully understood. In the PFC, we found that cocaine decreased TrkB mRNA levels, an effect that was prevented by CSF1R inhibition. Cocaine administration decreases BDNF levels and synaptic density in the PFC, affecting cortico-striatal glutamatergic signaling. Furthermore, BDNF microinfusion in this region can restore this signaling and reverse behavioral alterations induced by cocaine^86–90^. In this sense, the reversal of TrkB mRNA levels induced by the partial microglial depletion could contribute to the reduced behavioral sensitization.

The CSF1R pathway is involved not only in physiological, but also pathological conditions, being, therefore, an important target for the development of therapeutic approaches to treat numerous diseases^91^. The CSF1R signaling, via its both ligands, CSF1 and IL-34, regulates microglia development, function and viability^92,93^. However, there are some differences in terms of actions of these both agonists. CSF1 and IL-34 bind to different domains of the CSF1R^92^ and also may possess different functions in the brain^93,94^. Moreover, there are regional and cell differences in terms of their expression^94,95^. Finally, although CSF1 is mainly an agonist of CSF1R, IL-34 binds to other targets, such as the receptor type protein-tyrosine phosphatase-zeta and the transmembrane heparin sulfate proteoglycan syndecan-1, which regulate important cellular processes^96^. Nevertheless, some effects mediated by CSF1R in drug addiction could be due to its activation by IL-34. Thus, since in the present study we evaluated the effect of CSF1R inhibition in the behavioral alterations induced by cocaine, the role of the IL-34 in this pathway should be investigated in future studies.

This study has some limitations. CSF1R inhibition reduces the behavioral sensitization by cocaine, which represents only the binge/intoxication stage of neurobiology of drug addiction. Additionally, this work did not determine the cells responsible for the BDNF production. Since neurons and oligodendrocytes are important sources of BDNF, these cells may contribute to the alterations in the levels of this neurotrophic factor observed in the present study. Finally, although we found alterations in CX3CL1-CX3XR1 levels, which is a more specific microglia-neuron signaling, further studies are needed to elucidate the role of this signaling in the context of behavioral and neural alterations induced by cocaine.

## Conclusion

In summary, our results suggest the participation of CSF1R and microglia in behavioral sensitization induced by cocaine. In addition, PLX3397 changed CX3CL1-CX3CR1 and BDNF-TrkB levels, two effects that could be involved in the protective effect of CSF1R inhibition and that deserve further investigation. This study opens a new avenue to the investigation of the role of microglia in drug addiction.

## Material and methods

### Animals

All procedures used in this study were carried out in accordance with the guidelines of the Brazilian National Council of the Control of Animal Experimentation (CONCEA) and approved by the Ethics Committee on Animal Use of Federal University of Minas Gerais under the protocol number 231/2018. This study was performed in accordance with the ARRIVE guidelines. Experiments were conducted using male Swiss mice (8–9 weeks of age) obtained from Animal Care Facilities of the Institute of Biological Sciences (ICB). Animals were kept under controlled room temperature (24 °C) under 12 h:12 h light–dark cycle and ad libitum access to food and water.

All procedures used in this study were approved by the Ethic Committee on Animal Use of Federal University of Minas Gerais and institutionally approved under protocol number 231/2018. This study was performed in accordance with the ARRIVE guidelines.

### Drugs

In order to perform the study, the following substances were used: cocaine (Merck & Co., Inc.) diluted in saline (NaCl 0.9%); pexidartinib (PLX3397—MedChemExpress) diluted in dimethyl sulfoxide (DMSO) 1%, polyethylene glycol 300 (PEG-300—Synth) 45%, tween 80 5% and saline; ketamine 10% and xylazine 2%.

### Behavioral tests

#### Behavioral sensitization

Acute administration of psychostimulant drugs, such as cocaine, promotes behavioral changes like the increase in locomotor activity. In addition, repeated administration of the same dose of the drug over the days can promote plastic changes that accentuates this hyperlocomotion^38,97^.

In the behavioral sensitization test, animals were submitted to the open field to assess locomotor activity. The equipment consists of a circular arena of approximately 120 cm in diameter, surrounded by a circular wall of 45 cm height. Mice were placed individually at the center of the arena and the total distance traveled for 30 min was evaluated for 7 days^29,97^. In the first two days, animals were habituated to the arena and from the 3rd until the 7th day, animals were treated with intraperitoneal (i.p.) injection of cocaine (15 mg/kg) or saline. To test our hypothesis, animals were treated with PLX3397 (40 mg/kg)^37,98^, an CSF1R inhibitor drug, or vehicle by oral gavage once a day. In order to achieve a reduction of the microglia population at the beginning of cocaine injection, we started the administration of PLX3397 3 days before the first injection of the psychostimulant. In this protocol, PLX3397 treatment lasted 7 days (Fig. 1A). Animals were euthanized immediately after the end of the behavioral tests to collect the brain tissues for morphological and biochemical analysis.

#### Conditioned place preference

The paradigm of conditioned place preference (CPP) is based on Pavlovian conditioning, in which an environmental clue is associated with a stimulus Thus, this model is used to understand the rewarding mechanisms of a drug^38,97^.

CPP test consists of three phases: pre-test, conditioning and test^97,99^. In the pre-test (day 1), animals were placed individually in the central chamber (9.5 cm long, 5 cm wide and 12 cm height) in an acrylic box containing two other sides of equal dimensions (15 cm in length, 12 cm in width and 12 cm height). They were able to freely explore the three compartments. The pretest was recorded and the time spent in each compartment for 15 min was evaluated using the ANY-maze software. Mice that spent more than 70% of their time exploring one side were excluded from the next analysis. Conditioning phase lasted 6 days, and the animals were treated with cocaine or saline on alternate days in one side of the box, as follows: (I) groups treated with cocaine. On days 2, 4 and 6, animals were treated with cocaine (15 mg/kg, i.p.) and confined to one side of the box (drug-paired side), where they remained for 30 min. On days 3, 5 and 7, animals were treated with saline and confined on the opposite side. (II) groups treated with saline. Control groups were treated with saline solution every day and confined to a different side of the box after each injection. Animals were randomly positioned in each side of the after receiving cocaine. On the test day (day 8), animals were placed in the central chamber and were able to freely explore the environment for 15 min. The test was recorded and the exploration time of each compartment was evaluated. The result was presented as the preference rate (s) and the CPP score was defined as the time spent in the drug-paired chamber on the test day minus the time spent on that same side on the day of pre-test. To test our hypothesis, animals were treated with PLX3397 (40 mg/kg, p.o.) or vehicle once a day. In order to achieve a reduction of the microglia population at the beginning of cocaine injection, we started the administration of PLX3397 3 days before the first injection of the psychostimulant. In this protocol, PLX3397 treatment lasted 9 days (Fig. 1C). Animals were euthanized immediately after the end of the behavioral tests.

### Intracardiac perfusion and brain slice preparation

At the end of each behavioral test, animals were anesthetized with ketamine (80 mg/kg, i.p.) and xylazine (8 mg/kg, i.p.), and then were subjected to thoracotomy to expose the heart. Subsequently, a hypodermic needle connected to an infusion system (peristaltic pump) was inserted in the left ventricle, allowing the exchange the blood with a phosphate buffer solution in saline (PBS—pH 7.4), an infusion rate 4 mL/min. After infusing about 30 mL of PBS, the animals were perfused with a 4% paraformaldehyde solution (PFA—pH 7.4). After completing the perfusion, animals were decapitated, their brains were removed and stored in buffered PFA 4% overnight. Subsequently, brains were moved to a 30% sucrose solution, until complete saturation, then were frozen in isopentane 99% and dry ice for 20 s and stored at − 80 °C. Brains were sliced into 30-μm-thick sections at − 20 °C with the cryostat.

### Immunofluorescence analysis of Iba-1^+^ and CD68^+^ cells

In order to quantify the number of microglial cells and a suggestive activation state, slices (30 µm) were incubated with a citrate buffer for 60 min at 70 °C for antigenic recovery and washed with TBS and blocked for 2 h with blocking solution (4% BSA in 0.5% TBST). Slices were then incubated overnight with primary antibodies rabbit anti-Iba-1 (1:500; Wako Chemicals, Osaka, Japan) and rat anti-CD68 (1:500; AbD Serotec, Hercules, USA). Thereafter, slices were washed with TBS and incubated for 2 h with secondary antibodies goat anti-rabbit (1:1000; Alexa Fluor 594, Life Technologies, Carlsbad, USA) and goat anti-rat (1:1000; Alexa Fluor 488, Life Technologies, Carlsbad, USA). Immediately after, they were washed, incubated with DAPI 1.75 μg/mL for 30 min, washed again and mounted in gelatinized slides with Fluoromount (Sigma-Aldrich, St. Louis, USA). Slices were observed under fluorescence with LSM880 Zeiss microscope in 20X magnification. Since there are anatomical and functional differences between the NAc core and shell^100^, and both regions also present distinct roles in the context of addiction^101,102^, which could lead to different microglial responses^103^, both regions were separately evaluated. The Paxinos and Watson mouse brain atlas was used to localize and differentiate these nuclei. The quantification of labeled cell-bodies was performed using the software ImageJ 1.52a and the results are represented as the number of positive cell-bodies per field for Iba-1^+^ and total area stained for CD68^104^.

### Morphological analysis of microglia

In order to evaluate whether cocaine and/or PLX3397 affect microglial morphology, both Sholl analysis and cell body to total cell size ratio were applied. To this end, slices (30 µm) were first submitted to immunohistochemistry. Sections were incubated with citrate buffer for 60 min at 70 °C, pretreated with 1% H_2_O_2_ for 15 min and blocked for 2 h with blocking solution (4% BSA in 0.5% TBST) and incubated with rabbit anti-Iba-1 (1:500; Wako Chemicals, Osaka, Japan) during 72 h. After that, slices were washed and incubated with secondary antibody [biotinylated goat anti-rabbit, 1:1,000, VECTASTAIN Elite ABC Kit (Rabbit IgG)—VECTOR Laboratories, Burlingame, CA, USA). The sections were then incubated for 1 h with avidin–biotin peroxidase complex (VECTASTAIN ABC kit, Vector Laboratories, Burlingame, USA) at room temperature. Labeling was visualized by using diaminobenzidine (DAB) solution activated with 0.1% H_2_O_2_. Slices were observed with Zeiss Imager A.2 microscope in 40 × magnification lens and pictures were taken of the NAc core and shell, and also CA1 according to the Paxinos and Watson mouse brain atlas.

For Sholl analysis, 5 cells per animal from 5 animals per group were selected. Only individualized cells with complete dendritic trees were selected for analysis. For each animal, the mean value between the 5 cells were represented. After selecting microglia, adjusted threshold was applied to 8 bit images, followed by the application of the Sholl analysis plugin of Image J software. Concentric circles, centered on the soma and increasing 4 μm with every circle, were drawn. The number of intersections per radius, total intersections for each cell and the maximum branch length (based on the maximum radius intersected) were considered for evaluation. For generating cell body to total cell size ratio, adjusted threshold and analyze particles functions of ImageJ software were applied to 8 bits images from the three regions evaluated in the present work (3 pictures per region). The intensity threshold (default algorithm) and size filter were applied to measure the pixels from the cell body size and pixels from the total cell size (cell body + dendrites) as previously described^42,43^. Sholl analysis and cell body to total cell size ratio were utilized as a measurement for morphological changes that suggest microglial activation^40–43^.

### GFAP immunofluorescence

In order to estimate the number of astrocytes, slices (30 µm) were incubated with a citrate buffer for 60 min at 70 °C for antigenic recovery and washed with TBS and blocked for 2 h with blocking solution (4% BSA in 0.5% TBST). After that, slices were incubated overnight with primary antibody rabbit anti-GFAP (1:800; Cell Signaling, Danvers, USA). Then, slices were washed with TBST and incubated for 2 h with secondary antibody goat anti-rabbit (1:1000; Alexa Fluor 488, Life Technologies, Carlsbad, USA). Slices were washed and mounted in gelatinized slides with Fluoromount (Sigma-Aldrich, St. Louis, USA). Slices were observed under fluorescence with LSM880 Zeiss confocal microscope in 20X magnification lens and pictures were taken of the nucleus accumbens (NAc) core and shell according to the Paxinos and Watson mouse brain atlas. The quantification of labeled cells was performed by the software ImageJ 1.52a and the results are represented as total area stained for astrocytes^104^.

### Assessment of neurotrophic factors and CX3CL1

Brain samples from animals that have not been subjected to intracardiac perfusion were used in biochemical and molecular analysis. The prefrontal cortex (PFC), hippocampus and striatum previously dissected from animals submitted to behavioral sensitization were homogenized in an cytokine extraction solution [Tris–HCl 2 mM pH 8.0; 137 mM NaCl; 1% NP40; glycerol 10%; 0.1 mM phenylmethylsulfonyl fluoride (PMSF); 1 µM pepstatin A; 10 mM EDTA; E-64 10 μM and 0.5 mM sodium vanadium diluted in distilled water] and centrifuged at 4 °C at 14,000 rpm for 20 min. Subsequently, the enzyme-linked immunosorbent assay (ELISA) was performed to detect the concentration of neurotrophic factors (BDNF, NGF, GDNF) and chemokine (CX3CL1) using kits from R&D Systems (DuoSet, Minneapolis, MN), according to the procedures described by the manufacturer. Results are expressed as pg/µg of protein.

### Real time quantitative PCR (RT-qPCR)

Total RNA was obtained from the PFC, striatum and hippocampus from animals submitted to behavioral sensitization using TRIzol reagent (Life Technologies, ThermoFisher Scientific, MA, USA) according to manufacturer’s instructions. One microgram of RNA was used to synthesize first strand cDNA, prepared with SuperScript III reverse transcriptase (Invitrogen, ThermoFisher Scientific, MA, USA) following the manufacturer’s protocol. Quantitative PCR was performed using RT^2^ SYBR® Green qPCR Mastermix kit (Qiagen) with a CFX96 Touch™ Real Time detection system (BioRad). The primer sequences used (sense and antisense)were: *Cx3cr1* (TGCCTTCTTCCTCTTCTGGA; TAAAGGGGTTGAGGCAACAG), *Ntrk2* (CCGCTAGGATTTGGTGTACTG; CCGGGTCAACGCTGTTAGG) and *Rpl32* (GCTGCCATCTGTTTTACGG; TGACTGGTGCCTGATGAACT). The expression data were calculated as 2 to the power of − ΔCt (where ΔCt is the difference between Cts of the target gene and *Rpl32* reference gene). Results are presented as normalized values relative to saline + vehicle group.

#### Statistical analyses

A statistical analysis was performed using the statistical program Prism 8.0 (GraphPad, CA, USA). Behavioral, histological and biochemical data were submitted to the Kolmogorov–Smirnov test for normality evaluation and were subsequently analyzed using the two-way ANOVA followed by Tukey post-hoc test. Data were presented as mean ± standard error of the mean. The level of significance was set at p ≤ 0.05.

## Supplementary Information

Supplementary Figure 1.

Supplementary Figure 2.

Supplementary Figure 2 legend.

Supplementary Table 1.
