# Supplementary figures and images for: Inhibition of CSF1R, a receptor involved in microglia viability, alters behavioral and molecular changes induced by cocaine
Source: Sci Rep. 2021 Aug 6;11:15989. doi: 10.1038/s41598-021-95059-7 (PMC8346567; doi:10.1038/s41598-021-95059-7)

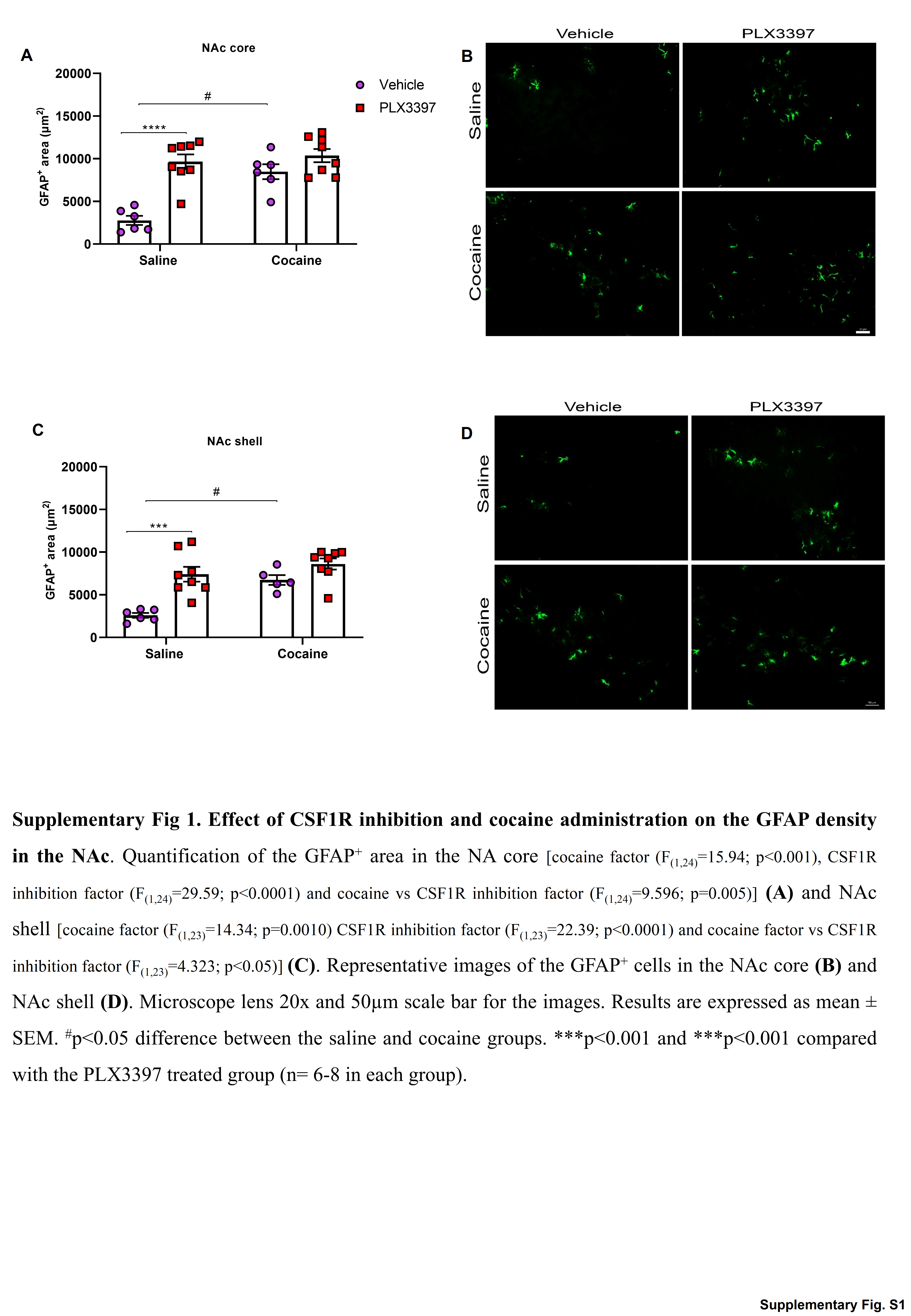

Supplement: Supplementary file 1 — Supplementary Figure 1. [file 41598_2021_95059_MOESM1_ESM.tif]

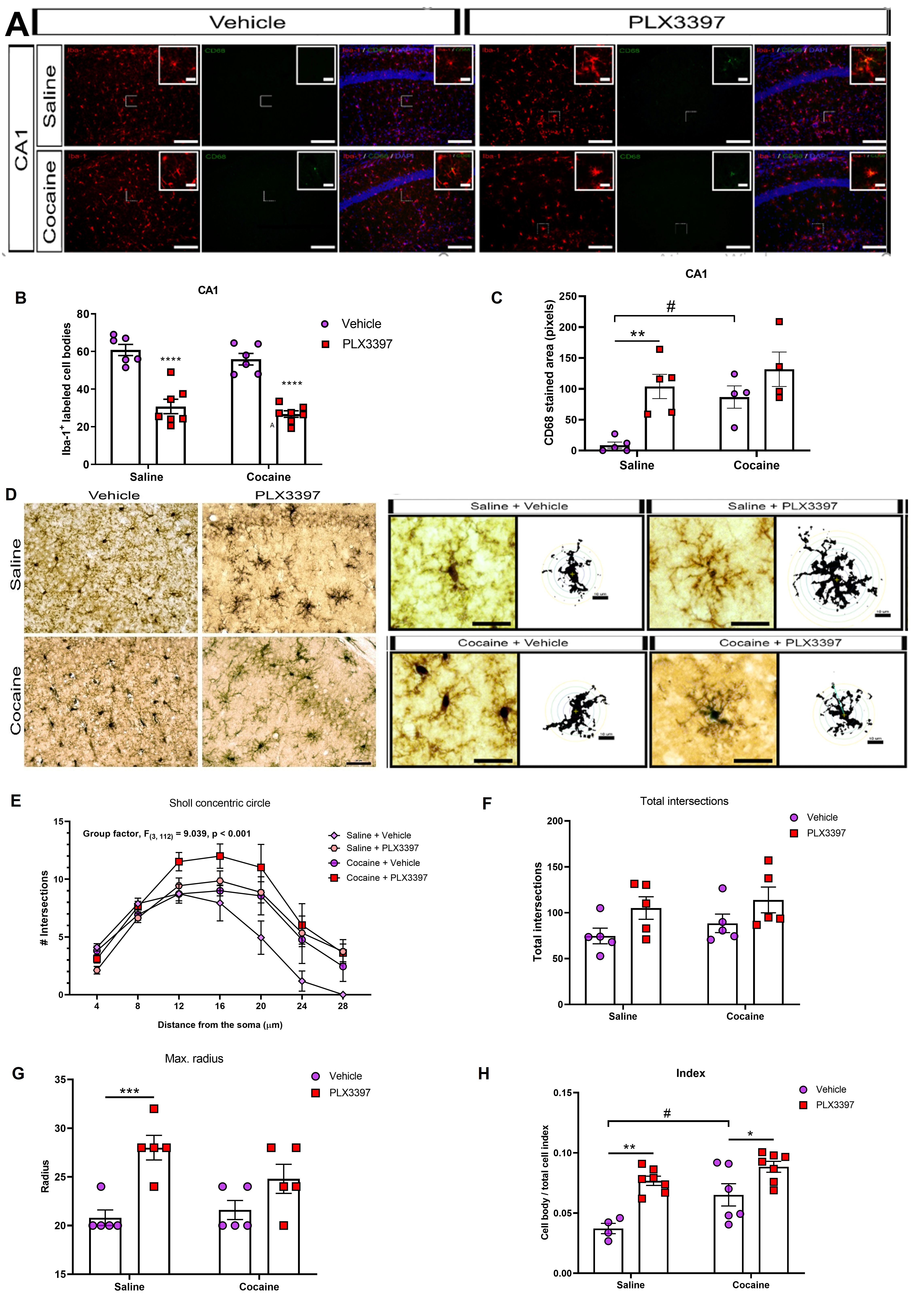

Supplement: Supplementary file 2 — Supplementary Figure 2. [file 41598_2021_95059_MOESM2_ESM.tif]

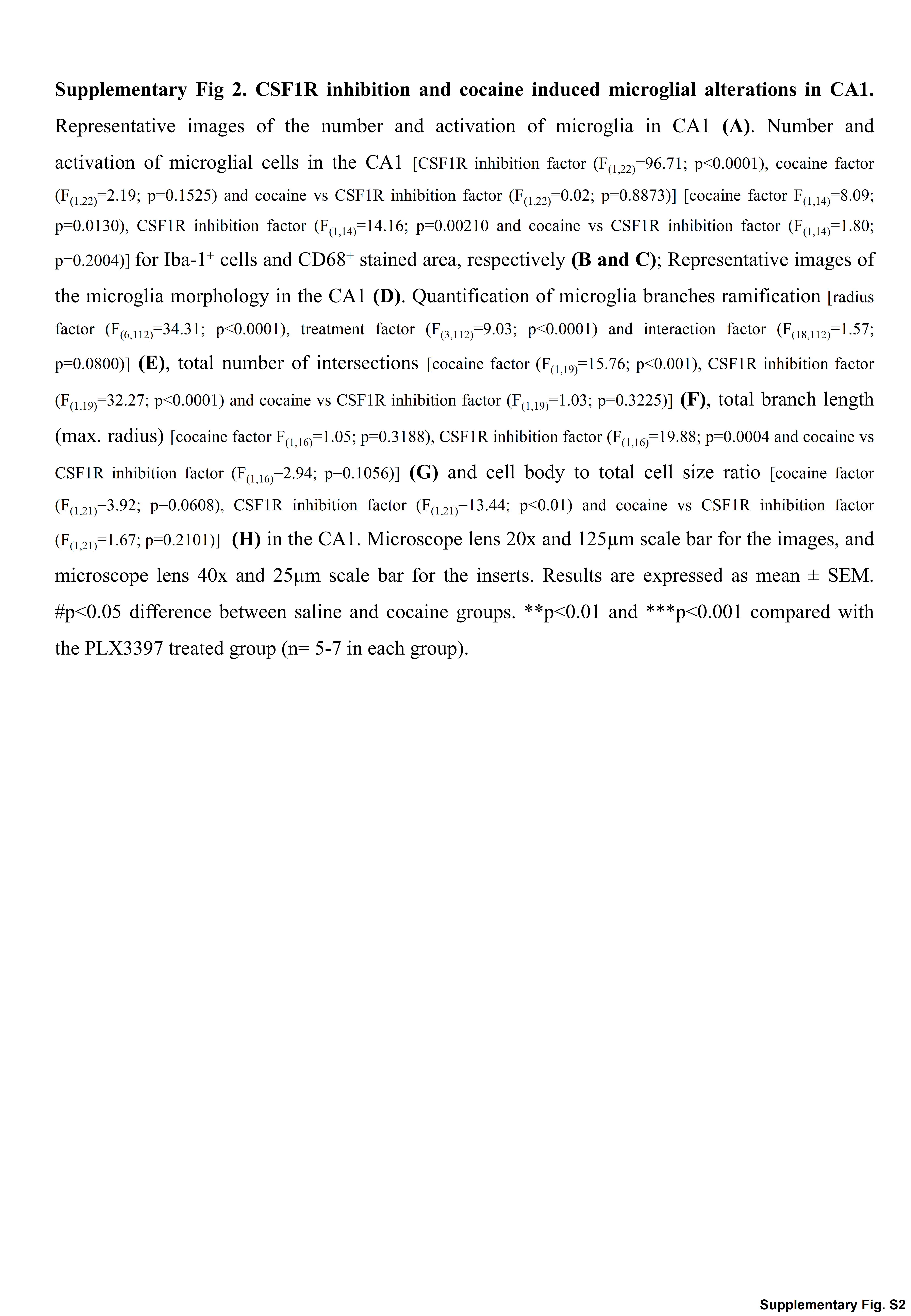

Supplement: Supplementary file 3 — Supplementary Figure 2 legend. [file 41598_2021_95059_MOESM3_ESM.tif]
