# Supplementary material for: Inhibition of CSF1R, a receptor involved in microglia viability, alters behavioral and molecular changes induced by cocaine
Source: Sci Rep. 2021 Aug 6;11:15989. doi: 10.1038/s41598-021-95059-7 (PMC8346567; doi:10.1038/s41598-021-95059-7)
Supplement: Supplementary file 4 — Supplementary Table 1. [file 41598_2021_95059_MOESM4_ESM.pdf]

Supplementary Table 1						
	Cocaine factor		PLX3397 factor		Interaction	
**GDNF** *Striatum*	$F_{(1,21)}$	p	$F_{(1,21)}$	p	$F_{(1,21)}$	p
Two-way ANOVA	0.76	0.3920	0.18	0.6720	1.10	0.3051
**GDNF** *HIP*	$F_{(1,22)}$	p	$F_{(1,22)}$	p	$F_{(1,22)}$	p
Two-way ANOVA	2.04	0.1666	0.59	0.4489	0.36	0.5501
**GDNF** *PFC*	$F_{(1,22)}$	p	$F_{(1,22)}$	p	$F_{(1,22)}$	p
Two-way ANOVA	12.46	0.0019	0.36	0.5498	0.30	0.5891
**NGF** *Striatum*	$F_{(1,23)}$	p	$F_{(1,23)}$	p	$F_{(1,23)}$	p
Two-way ANOVA	0.02	0.8870	0.001	0.9696	2.04	0.1665
**NGF** *HIP*	$F_{(1,20)}$	p	$F_{(1,20)}$	p	$F_{(1,20)}$	p
Two-way ANOVA	0.002	0.9872	0.005	0.9438	0.31	0.5827
**NGF** *PFC*	$F_{(1,15)}$	p	$F_{(1,15)}$	p	$F_{(1,15)}$	p
Two-way ANOVA	7.19	0.0171	0.03	0.8449	3.21	0.0930

Abbreviations: HIP: hippocampus; NA core: nucleus accumbens core; NA shell: nucleus accumbens shell; PFC: prefrontal cortex
